# Prospects of Observing Ionic Coulomb Blockade in Artificial Ion Confinements

**DOI:** 10.3390/e22121430

**Published:** 2020-12-18

**Authors:** Andrey Chernev, Sanjin Marion, Aleksandra Radenovic

**Affiliations:** Laboratory of Nanoscale Biology, Institute of Bioengineering, School of Engineering, EPFL, 1015 Lausanne, Switzerland; andrey.chernev@epfl.ch (A.C.); sanjin.marion@epfl.ch (S.M.)

**Keywords:** nanofluidics, ionic Coulomb blockade, 2D materials, nanopores, nanotubes, angstrom slits

## Abstract

Nanofluidics encompasses a wide range of advanced approaches to study charge and mass transport at the nanoscale. Modern technologies allow us to develop and improve artificial nanofluidic platforms that confine ions in a way similar to single-ion channels in living cells. Therefore, nanofluidic platforms show great potential to act as a test field for theoretical models. This review aims to highlight ionic Coulomb blockade (ICB)—an effect that is proposed to be the key player of ion channel selectivity, which is based upon electrostatic exclusion limiting ion transport. Thus, in this perspective, we focus on the most promising approaches that have been reported on the subject. We consider ion confinements of various dimensionalities and highlight the most recent advancements in the field. Furthermore, we concentrate on the most critical obstacles associated with these studies and suggest possible solutions to advance the field further.

## 1. Introduction 

**Figure 1:**
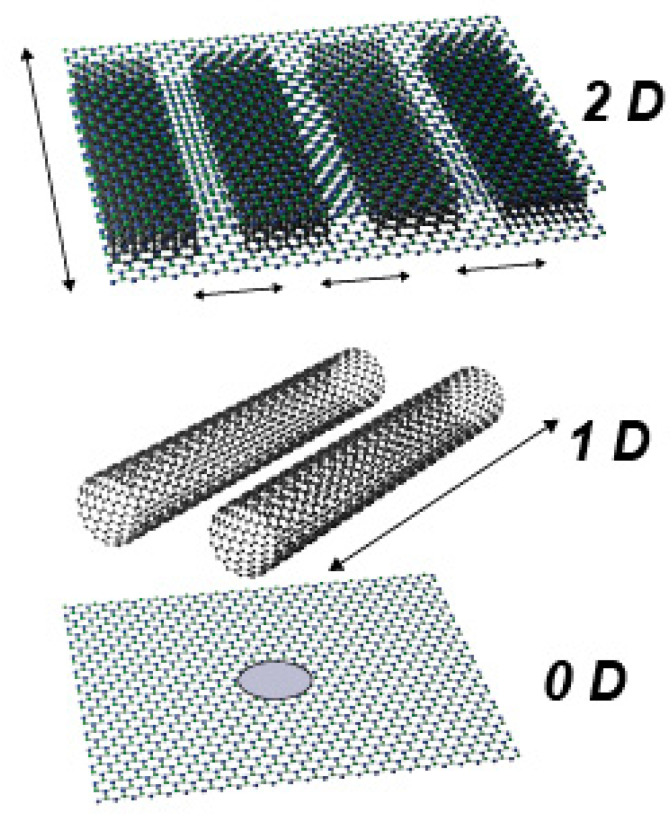
Different geometries are considered for confined nanofluidic systems from two-dimensional (2D) nanoslits to 1D nanowires and quantum dot-like nanopores in the atomically thin material matrix, i.e., 2D nanopores.

In the past fifteen years, various artificial nanofluidic platforms have become highly compelling for fundamental studies of physical phenomena and numerous practical applications where the transport of the confined ions plays a crucial role [1,2]. Among the most exciting practical applications are power generation [3,4,5,6,7], filtration, and molecular separation [8,9,10]. The last five years have witnessed remarkable progress in the fabrication of nanofluidic devices, enabling researchers to develop artificial nanofluidic systems with the confinement of one to a few water molecules (below 1 nm) [11]. Such nanofluidic platforms have been realized in zero-dimensional (0D), 1D, or 2D geometry [7,12,13,14] (Figure 1**)**. These platforms exhibit giant permeability and ion selectivity comparable to biological ion channels, excluding anions and macromolecules, and closely mimic functionalities previously observed only in biological channels. Superb selectivity of the sodium/calcium family of channels has fascinated scientists due to its physiological relevance and underlying physical mechanism [15,16,17]. A simplified electrostatic and Brownian dynamics model of the prototypical model Ca^2+^ or Na^+^ channel has been used in describing its conduction and selectivity [18], echoing the phenomenology of Coulomb blockade [17]. The term ionic Coulomb blockade (ICB) was suggested first as a counterpart of the electronic Coulomb blockade (ECB) by Krems and Di Ventra [19]. Confinement below 1 nm dictates a departure from the mean-field assumptions as the correlations between the ions and finite-size effects cannot be neglected and complicates the insight into the ionic charge transfer at the nanoscale [17,20,21]. 

Artificial nanofluidic devices developed for single-ion conductivity measurements provide a unique opportunity to test the proposed models and reveal the many-body effects in ionic systems. This perspective article aims to discuss practical challenges in verifying the models that evoke ionic Coulomb blockade in a variety of settings that are now available due to recent progress in nanofabrication [12,22,23]. Furthermore, it also provides suggestions for the integration of additional approaches such as modulation of the charges, pressure, pH, ionic strength, temperature, and potential in the range not attainable on lipid bilayers. 

As suggested initially, ionic Coulomb blockade is based on the relation of ion kinetic and barrier energies and manifests as the nonlinear transport of ions. This behavior was later experimentally observed in atomically thin, sub-nanometer-sized molybdenum disulfide (MoS_2_) nanopores [13]. Although theoretical models predict ICB and suggest particular conditions to reveal the effect [17,20,24], irrefutable experimental observation of this phenomenon is still challenging. The reason for that is a whole set of issues that have to be taken into account before considering this complex process. The most critical issues that could mask ICB observation are related to the fact that ionic transport is measured at room temperatures, causing large charge fluctuations, which leads to the increased noise, and instability of the nanofluidic devices. Together with regular wetting and contamination challenges in nanofluidics [25], we can argue that the ionic systems’ nonlinear current-voltage characteristics are insufficient to prove the ICB effect. Unlike in the case of ECB [26], there are still no convincing data showing conductance oscillations or single-ion devices.

Therefore, it is essential to critically assess the suitability of different nanofluidic platforms for the observation of ICB and propose novel solutions that will allow us to untangle the role of the issues mentioned above. New geometries like nanopores in 2D materials, single-digit nanotubes and angstrom slits play a key role and are essential in verifying the different parameters required to observe ICB reproducibly. The complex nature of ionic transport can be considered with different settings and thus allow direct observation of experimental parameter variation such as pore length, diameter, access resistance, surface charge, gating voltage temperature, and many others [27]. 

## 2. Prerequisites for Ionic Coulomb Blockade

The basic prerequisites for ionic Coulomb blockade are shared with its electronic counterpart [28,29]. The geometrical requirement for ICB to occur is represented by two reservoirs with charge carriers, in our case ions, with a channel in between with a large resistance to ion transport at least in one direction [17,19,20,24]. More specifically, the channel has to provide the type of confinement that allows ions to dwell inside of it as it was suggested originally [15]. Ion transport between the two reservoirs is inhibited due to strong Coulomb interactions and an ion dwelling at or near the channel, so that there is a limit on the possible charge, which can be transferred between the two sides of the chamber [17,19,20,24]. When the energy barrier for ions to enter the channel, and subsequently traverse it, is larger than the system’s thermal energy, ionic Coulomb blockade can be observed. We identify two main mechanisms that are expected to result in ionic Coulomb blockade signatures in nanopore or nanochannel systems. One is linked to capacitive charging [19,30] and analogous to the electronic case of a tunnel junction. The second mechanism is linked to the existence of an “island” corresponding to a quantum dot in ECB, which can be gated [15,16,20] and can be further developed towards the analogy of the single-electron transistor [20].

The first case, which we call the capacitive ICB, is relatively similar to the ECB case with a single tunneling junction. It occurs if an ion transits between the two reservoirs and charges it analogously to a capacitor, thus producing a barrier for further ions to transit [19,30]. 

This case requires that the channel/pore be ion-selective so that a capacitive barrier can form for an ion of valence z, that the thermal energy is lower than the capacitive self-energy of the channel $U=Q^{2}/2C_{s}$, and that the transferred charge Q=nze dwells inside the pore or next to the pore entrance for a sufficiently long time for it to interact with the transport of further ions. We note that the ICB effect is expected to be stronger for ions carrying more than one unit of charge, as the self-energy has a quadratic dependence on the valence *z.* The capacity of the channel/pore of length L and radius R to store charge is the self-capacitance $C_{s}$ and can, neglecting any fine effects, be approximated by $C_{s}=4\pi \epsilon_{0}\epsilon_{r}R^{2}/L$, with $\epsilon_{r}$ the relative dielectric constant of water. In general, the capacitive self-energy is a good measure if ICB is detectable as it gives information on the resulting energy barriers for ion transport.

Coarse-grained molecular dynamics simulations [19,30] indicate capacitive ICB could occur in sub-nm pores in quasi-2D or 2D materials but also predict a weak nonlinear current-to-voltage dependence uncharacteristic for the classical electronic Coulomb blockade. In contrast, Brownian dynamics simulations show that the role of screening and ion-pair formation via Bjerrum pairs are paramount in these systems [20]. Here, both parameters are strongly modulated by the dielectric constant of water, which has been shown to be reduced in confinement in respect to its bulk value [31,32], and which would decrease the capacitive self-energy and require lower temperatures or smaller and thinner pores for ICB to be detectable. Moreover, finite-size effects would need to be considered, including the peculiarities of pore-wall interactions, which can strongly influence ion transport in quasi-2D or 2D membranes [33,34,35,36].

In the second case, ionic Coulomb blockade happens in the systems that are reminiscent of a quantum dot, confined with two energy barriers—schemes that are used for ECB observations and applications. As for the ICB counterpart, it can be observed in the setting where the ion occupancy of the channel limits ion transport [17,20] and where it has a role analogous to an isolated quantum dot in the electronic case [29]. Here, ions need to have an energetically favorable position inside the channel, which binds them there in spite of thermal motion so that their presence would block other ions traversing the channel. This stable position can be formed due to ion-channel interactions, either by electrostatic gating voltage modulation or by the presence of surface charges [24,37]. In the latter case, the surface charge will then attract oppositely charged ions and thus block ion transport through the channel.

When a fixed voltage bias is applied between the reservoirs, the ion current oscillates with peaks at certain quantized values of these gating surface charges. A typical signature of ICB is when this neutralizing charge has a stepwise dependence on the gating voltage or surface charge, and when the conductance of the channel has peaks at certain values with conductance almost completely suppressed otherwise [17].

Microscopically, there are several distinctions between the ionic and the electronic Coulomb blockades. While electrons are negatively charged by default, ions can be both negative and positive, with different valences available. These ions are also present in a solution so that the electrostatic interaction between them is screened, losing its long-range components. Thus, such an effect will be local and will require extreme confinements for size exclusion and Coulomb interactions to dominate transport properties. In the case of strong 1D confinement, ion transport through such channels is expected to go through the dissociation of pairs of ions (second Wien effect) [38,39].

Nevertheless, in the case of gating via voltage or wall charge inhibiting the movement of ions due to tight binding, some of the ions are tightly bound, and only under certain conditions are they able to dissociate, a situation recently termed as a “fractional Wien effect” [20]. Under this model, it is also predicted that when electrostatic screening becomes too large, the ionic Coulomb blockade effect becomes suppressed. This also provides an upper limit on the size of a confining channel to about 2.5 nm. This means that, in certain geometries, ICB could be detected even in pores larger than one nanometer, depending on the magnitude of electrostatic screening.

Providing unquestionable proof for the ionic Coulomb blockade is not an easy task. Most experimental “wet” implementations cannot easily change the interaction between the ions and the channel interior, i.e., by changing its surface charge or applying a gating voltage to it. One of the major indicators of ICB is nonlinear current-to-voltage curves [17,20,24,35]. As the bias voltage is increased until a certain threshold is crossed, there is no ion transport through the system. After that, a nonlinear increase in current is expected until linear (ohmic) behavior is obtained. In the classical electronic CB, several steps called the Coulomb staircase in the current versus voltage curves are expected in this nonlinear regime [29]. Recent work indicates that, in the case of ICB, there could be no such steps, with a direct transition to an ohmic regime [20]. However, it is problematic to identify ICB only with current-voltage characteristics (IV curves), especially in the absence of a staircase-like pattern. Realistic devices are known to exhibit leakage currents that will exceed the current in the blocked ICB conditions [13], possibly overshadowing any conduction steps.

Furthermore, similar nonlinear IV curves are known to occur during the electrowetting of channels and are caused by remnant gas inside the pore system [25,40,41,42]. Partial wetting of these systems presents with nonlinear activation-like IV curves, similar to the ones that have been associated with ionic current blockade [13]. There are many open questions regarding our understanding of ICB, which could help to design better experiments. Variations of the ambient temperature could be used to probe the activation energy barriers and possibly promote ICB. Varying the solution’s pH could be used to change the gating charges, thus enabling the variation of the gating charge in larger channels. However, the main requirement is still to manufacture a device with such properties to optimize the conditions for the observation of ICB, a topic we tackle in the following sections.

## 3. ICB in 2D Nanopores

Over the last decade, 2D materials have become a rich area of research and are showing tentative signs of impacting our everyday life [43]. In bulk, these materials have the form of layered crystals, with van der Waals interaction holding together 2D layers with a thickness starting from 0.3 nm. These 2D sheets are under intense study because of their fascinating electronic properties, spanning the range from isolating and semiconducting to superconducting. Nanopores formed in 2D membranes from graphene [44,45,46], hexagonal boron nitride (h-BN) [47], transition metal dichalcogenides (TMDCs) [4,48], and MXenes [49] have been used to investigate nanofluidic phenomena or can act as a single molecular sensor.

2D nanopores have the peculiarity that due to their thickness, or lack of it, the pore sizes need to be small for the interactions between a single ion and the pore to dominate ion transport. For capacitive ICB to occur, we need ion selectivity, a sufficient capacitive barrier, and ions to remain next to the pore entrance for a sufficient time. 2D nanopores are known for their selectivity to ions, which is expected to come from the surface charges and the resulting ion enhancement zones near the pore [5]. Simple classical estimates of the self-capacitance would support 2D material nanopores having a sufficient energy barrier [13], but there is no evidence of ion retention for sufficiently long times to cause this blockade effect unless the ion is retained inside the pore itself. Classical bulk models for nanopore conductance break down as we approach the nanometer scale [21,33,50], requiring us to include the specifics of the pore atomic structure and its interactions with the ions and water.

Ion transport in 2D systems is a rich topic of research [33,51], but little is known experimentally about the peculiarities of transport in sub-nm pores. When the degree of confinement approaches the ion’s size, i.e., sub-nm size ranges, then an effect called hydration layer shredding occurs [19,37,52]. If an ion needs to lose a part of its hydration layer to traverse the pore, it experiences an energy barrier that can also result in a nonlinear IV curve, one of the expected signatures for ICB. Unless there is some sort of weakly bound state for the ion in the middle of the channel, this hydration layer shredding is not enough by itself to induce a Coulomb blockade effect. This can be achieved by tailoring pore interior bond edges, which could temporarily bind an ion inside the pore, causing it to block further ion transport [50]. Pore edge termination is known to influence ion transport [53,54,55] and could be tailored by binding other chemical species and modifying the pore interior [54,55,56,57,58]. Ion transport through pores is determined by bulk solution and solvent-mediated interactions between the ions and the pore interior edges. This interaction has been shown to cause ions to dwell inside the pore [24,35]; however, it is unclear if this influences ion transport via the Coulomb blockade mechanism.

**Figure 2:**
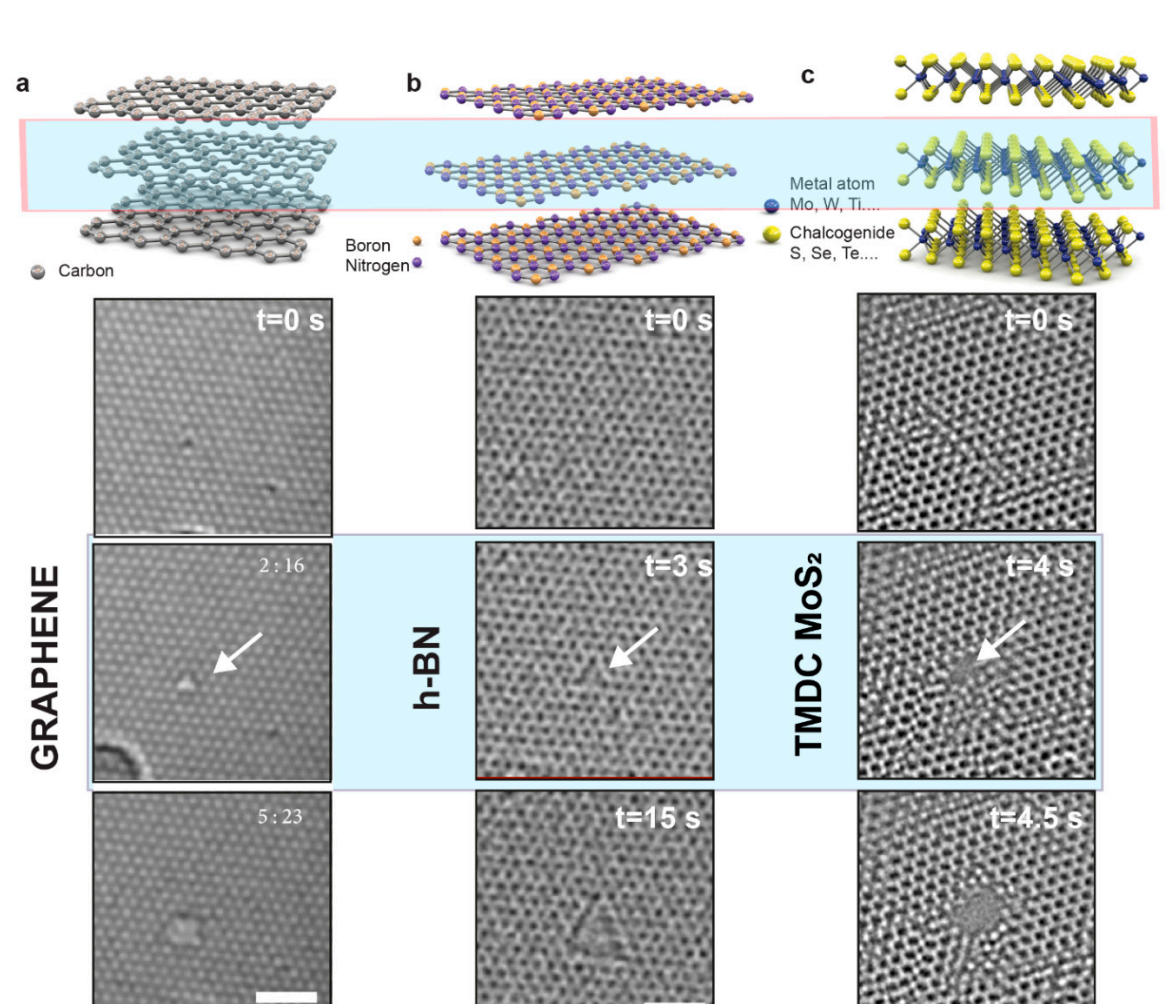
(**a**–**c**) Selection of the most popular 2D materials, i.e., graphene, hexagonal boron nitride, and molybdenum disulfide structures in bulk and monolayer form and generation and evolution of defects in 2D materials under e-beam. Defects and pores are created under 80 keV e-beam irradiation while imaging with a transmission electron microscope. Images adapted from (**a**) [59] and (**b**,**c**) [48].

2D nanopore systems have several unresolved issues that still require further study. Control of the pore shapes and sizes is not easy to achieve as pores are known to be highly sensitive to the fabrication method [47,59] (Figure 2). However, this can be addressed by the predefined pore shapes. Together with electrostatic gating, it is expected to be a highly effective approach for ion selectivity and controlled transport [24]. In this case, the trapped cations themselves not only act as natural clogs of the pores, but also “protect” the clogged pore states by creating the repulsive potential around each pore that essentially suppresses the knock-on mechanism by mobile cations dancing around.

Solvation of pore systems is expected to be able to change the pore structure, and etching of pores and their enlargement due to chemical species or dissolved reactive oxygen. In addition, 2D systems are prone to surface contaminants (e.g., hydrocarbons) that originate from the 2D material transfer process [60]. These contaminants can clog pores or change their properties, making controlled experiments difficult. Additionally, wetting or clogging issues can plague these systems [25] and possibly even produce nonlinear IV curves due to the presence of nanobubbles [40,41,61]. Moreover, the standard method of wetting all nanoscale devices uses prewetting with water-ethanol mixtures, a procedure known to produce nanobubbles on hydrophobic surfaces and which has been linked to the same effect on mildly hydrophobic 2D material surfaces [25]. In addition, remnants of bound ethanol, or other molecules, have been shown to modify surface properties and can persist after subsequent flushing [62]. Differentiating these effects from ICB nonlinear IV curves requires additional probes and separate gating control.

**Figure 3:**
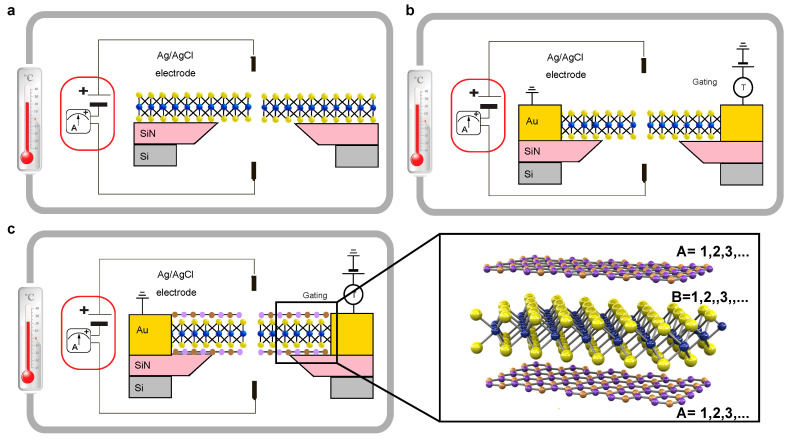
Common 2D nanopore measurement system with temperature control (**a**) equipped with transverse contacts for additional measurement channel or gating (**b**) and 2D material encapsulation (hBN-MoS_2_-hBN) (**c**) for the improved signal-to-noise ratio and decoupling of the measurement circuits (cis-trans and transverse).

Gating can be applied to nanofluidic platforms for modulating ICB in 2D pores (Figure 3). Notably, it can be applied in various ways, and not only limited to electrostatic gating. For example, one approach would be applying mechanical stress directly to nanopores [63] as the strain has been predicted to modulate ion transport through sub-nm 2D nanopores by promoting or removing a stable dwelling spot for ions in the center of the pore [31,32,33]. Strain moves the pore edge atoms out of their equilibrium positions, changing ion–atom and ion-water interactions, thus modifying the free energy landscape for the ions, possibly promoting or destroying a bound state for the ion inside the pore. Chemical gating of these systems is limited, and the surface charge can be modulated via pH modulation [11] and photo-gating [6], but it is not expected to make significant changes to the interaction of ions with the pore edges and could promote pore growth due to etching. Another useful probe would be studying the effect of temperature variation to provide information about the energy barriers for transport and possibly promote or suppress ICB effects. Although promising, this strategy has a reduced temperature range, in essence, from the melting to the boiling point of the solution. 

### Heterostructures

A promising approach may be to use layered heterostructures of 2D materials [64]. Here, a stable position for an ion inside the pore could be designed through a combination of different materials in A-B or A-B-A scheme where the monolayers are held together by van der Waals forces (Figure 3c). The major challenge for this is the existence of hydrocarbon contaminants complicating the reproducible production of such heterostructures using material transfer, where one 2D material (B) is transferred over another (A). This disadvantage would not be an issue in the case of in situ growth of heterostructures. Similar heterostructures have already been opened in several different research avenues, such as graphene interacting with h-BN allowed several groups to study the Hofstadter butterfly effect while numerous optoelectronic devices were based on the heterostructures from semiconducting monolayers [65,66,67].

Van der Waals heterostructures could be used to achieve direct gating by sandwiching a semiconductor such as MoS_2_ in between two layers of a wider bandgap energy 2D material such as h-BN. This suggested geometry could be considered either on a stand-alone basis or in combination with the in-plane transverse gating similar to the previous nanopore field-effect transistor (FET) approaches [68,69].

Another promising setting could be achieved by using stacked membranes (i.e., graphene oxide), where the percolating path of ions through the porous membranes and between the layers could be modified due to ions getting stuck and blocking ion transport [70].

However, to progress in the aforementioned systems, one has to have solid fabrication approaches for each of them. It is important to advance in nanopore fabrication techniques to reliably produce sub-nm pores through layered 2D membranes using existing techniques, namely an electron beam in transmission electron microscope [50,60,61,71] or electron beam lithography [72,73]. This approach is known to be the most common practice in nanopore research. However, complex multiple-step processes often result in relatively high contamination and low device yield; thus, alternative solutions have to be applied. 

One possible solution is a 2D material growth over the aperture in a suspended SiN membrane [73]. However, the first attempts show that the SiN pore ends up inducing growth, and thus, the island of growth is located exactly above it. Another fine point is that the resulting 2D material might be polycrystalline, which makes its structure controversial for pore stability. Following that, this approach requires a significant improvement in order to benefit from the potential cleanliness of such a process.

On the contrary, the electrochemical reaction allows the precise control of the pore size in the flow cell [74]. However, this technique does not allow the pore size confirmation other than using ionic current-voltage characteristics. The reason for that is a poor quality of nanopore samples after the nanofluidic experiments, as they appear to be irreversibly contaminated after the measurements in a flow cell.

## 4. ICB in 1D Nanowires

**Figure 4:**
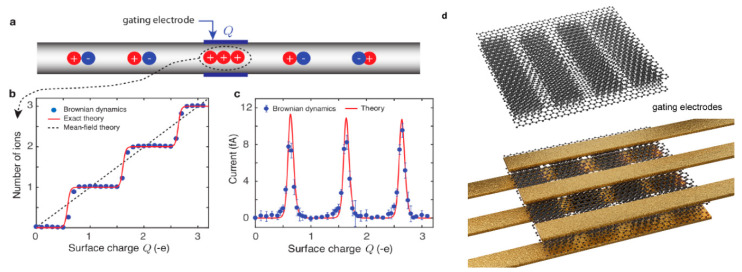
1D experimental geometry suggested for the ionic Coulomb blockade observation (**a**–**c**) in 1D nanochannels and (**d**) 2D nanoslits by the application of an external electric field and a consequent electrostatic gating of the ion-confining channel. Pictures (**a**–**c**) reprinted from Kavokine et al. [20].

The 1D channels represent the next promising nanofluidic platform (Figure 4). Such systems, reminiscent of narrow nanotubes, have been proposed as a very effective ion-confining solution capable of showing the single-ion transport and consequently able to reveal an ICB [20]. Furthermore, based on the geometrical confinement properties, Fermi-Dirac distribution for ions can be achieved, and thus, single-ion transport was predicted [17] and explained in detail for biological channels [17] and infinite 1D channels [20].

State-of-the-art techniques that allow single nanotube manipulation [3,75], if applied to the ultra-narrow nanotubes, could allow fabrication of a single conductive channel for ions, 1D ionic channels that show great potential for the observation of ICB. Inspired by ECB applications [76], 1D ionic channels were also suggested as the charge-carrier pumping systems, where the gating voltage on multiple gating contacts oscillates out of phase and thus allows a turnstile mechanism and a single-ion passage through the channel [20]. The unifying point of the aforementioned theoretical works [17,20] is the fixed wall charge that has to be controlled in order to manipulate the charge carrier transport at a single-ion level. However, despite the great success in nanofabrication [3,75], theoretically predicted effects are still to be confirmed experimentally, partly due to an even higher complexity of nanofabrication required (point contact, low leakage current, room temperature of measurements, high device yield). However, from the technical point of view, nanofabrication approaches developed for carbon nanotubes and nanowire field-effect transistors could be of help when applied to one-dimensional ion channels [75,77].

## 5. ICB in 2D Nanoslits 

First of all, 2D nanoslits inherited the flexibility of material stacking that was suggested for the first time in graphene research [66]. Following that, as in the case with 2D nanopores, this type of ion confinement in 2D nanoslits could benefit from a wide variety of available materials [59]. Furthermore, 2D nanoslits attract a particular interest as a first-ever system to experimentally show the value of the dielectric constant of water in confinement [32], frictionless water transport [78], and unmatched selectivity, allowing only water and protons to pass through the narrow 2D nanoslits [7]. However, despite the significant flexibility given by the fabrication process of 2D nanoslits, one particularly important property has to be considered—these systems are represented by multiple nanofluidic channels, and thus, read-out of a single-channel ion transport and control over the transport at a single-ion level is still complicated. 

One possible solution to that would be an extensive application of the most advanced fabrication techniques allowing the electrostatic control over each channel (Figure 4d) and thus, limiting the ionic current to a single channel of interest, a similar level of control could also be achieved mechanically similarly as in microfluidics [79]. Moreover, the 2D nanoslit fabrication approach [12] lacks control over channel side wall quality, and despite the atomically smooth surface of top and bottom of the channel, this might significantly affect the transport in narrow nanoslits with the comparable aspect ratio in both horizontal and vertical directions. Nonlinear transport of ions in such systems has been shown [14]; however, in that case, it is not associated with the Coulomb blockade of the channels due to a multiple input from a selection of nanoscale effects that might affect the conductance [80]. One possible solution to that would be an improved electrostatic control over the electric field in the channel as mentioned earlier (Figure 4d), similarly as proposed for other geometries. 

## 6. Summary

The complex nature of ion transport in nano-confined systems is usually challenged with natural artifacts, such as improper wetting of the pore, pore instability (especially in the case of a 2D nanopore), inability to confirm the pore size after the measurement, and many others. Development of a well-defined approach that would be able to address these issues one by one would propel the research field. However, this conception sounds much easier than it is; one would think of the experiments where the local temperature is controlled, together with the further gating of the confining geometry (either electrical or optical).

Temperature control is important in such systems since it has a significant impact on the ions’ mobility, the potential barrier size, and charge fluctuations, which might increase noise and the instability of nanofluidic devices. Therefore, in both ICB and ECB cases, the temperature criterion has to be fulfilled, i.e., *kT<<Ec.*

Electrostatic or optical gating of the confinement is expected to provide a crucial transition from a closed to an opened state within the system. The current status of nanofabrication allows us to create wafer-scale batches of devices showing a corresponding quality [22], and to combat one of the most important bottlenecks in nanopore research field—the small amount of nanopore devices and consequently low statistics of the measurements. Together with the demonstrated pioneer approaches [68,69,81] the nanopore research field is reaching the limitations and heading towards the achievement of the controlled confinement that would allow us to probe charged states within the pore on a single-ion level.

However, we assume that it is important to show the most controversial points that still have to be resolved in the field. For example, nanoscale contamination is starting to play an even more crucial role in nanoelectronic devices. Leakage currents and electrochemical reactions happening at the nanoscale in 2D materials and gating contacts of nanopore field-effect transistors come into play [68,69]. With irreproducible point-like defects and contaminants, ICB observation is even more challenging since, unlike in ECB, one cannot simply “freeze” the unwanted states and free charges. This problem keeps growing as long as we need more fabrication steps to produce a better-controlled ion-confining system.

Furthermore, recent studies [25] have shown that nanoscale contamination may lead to partial rewetting, which adds a high level of complexity for these studies. In particular, we have to admit that the most common practice of pore wetting with ETOH solution with subsequent electrolyte flushing and exchange may lead directly to the formation of nanoscale gas bubbles [61]. Apart from that, the typical ETOH wetting process has been shown to change the hydrogen distribution over the surface, and thus, change the ion-solvation part of the interactions of the ions with the surface and consequently mask the ICB.

Nevertheless, we would like to draw attention to the most promising future prospects and applications of this field to probe each contribution to ion transport phenomena and to improve the control over these platforms. That would allow us to achieve a synthetic model of a cell’s ion channels on a discrete channel level and benefit from step-by-step additions to a solid-state platform’s controls. As a result, we may expect to obtain crucial insights on gating, strain, temperature control, etc., to use as inputs in the bottom-up computation design of biopores [82], which have already proven themselves as a unique platform for sensing and single-molecule experiments [83]. So far, no further ICB signatures have been experimentally demonstrated as conductance oscillations and Coulomb staircase, even though these have been predicted for biological nanopores [17].
